# Changes and significance of vascular endothelial injury markers in patients with diabetes mellitus and pulmonary thromboembolism

**DOI:** 10.1186/s12890-023-02486-5

**Published:** 2023-05-26

**Authors:** Fan Li, Lianfang Yuan, Na Shao, Xiaokun Yang, Shaohua Yang, Linjia He, Jie Ding, Ming Ding, Shengzhe Yang, Wenwen Fu, Congcong Wang, Xiaochen Li, Qiling Cai

**Affiliations:** Respiratory Medicine Department, Chu Hsien-I Memorial Hospital and Tianjin Institute of Endocrinology, NHC Key Laboratory of Hormones and Development / Tianjin Key Laboratory of Metabolic Diseases, Tianjin Medical University, Tianjin, 300134 China

**Keywords:** Pulmonary embolism, Diabetes mellitus, Vascular endothelial injury, Soluble thrombomodulin, Von Willebrand factor, Circulating endothelial cells

## Abstract

**Background:**

To investigate the changes and clinical significance of vascular endothelial injury markers in type 2 diabetes mellitus (T2DM) complicated with pulmonary embolism (PE).

**Methods:**

This prospective study enrolled patients with T2DM hospitalized in one hospital from January 2021 to June 2022. Soluble thrombomodulin (sTM) (ELISA), von Willebrand factor (vWF) (ELISA), and circulating endothelial cells (CECs) (flow cytometry) were measured. PE was diagnosed by computed tomography pulmonary angiography (CTPA).

**Results:**

Thirty participants were enrolled in each group. The plasma levels of sTM (151.22 ± 120.57 vs. 532.93 ± 243.82 vs. 1016.51 ± 218.00 pg/mL, *P* < 0.001) and vWF (9.63 ± 2.73 vs. 11.50 ± 2.17 vs. 18.02 ± 3.40 ng/mL, *P* < 0.001) and the percentage of CECs (0.17 ± 0.46 vs. 0.30 ± 0.08 vs. 0.56 ± 0.18%, *P* < 0.001) gradually increased from the control group to the T2DM group to the T2DM + PE group. sTM (OR = 1.002, 95%CI: 1.002–1.025, *P* = 0.022) and vWF (OR = 1.168, 95%CI: 1.168–2.916, *P* = 0.009) were associated with T2DM + PE. sTM > 676.68 pg/mL for the diagnosis of T2DM + PE achieved an AUC of 0.973, while vWF > 13.75 ng/mL achieved an AUC of 0.954. The combination of sTM and vWF above their cutoff points achieved an AUC of 0.993, with 100% sensitivity and 96.7% specificity.

**Conclusions:**

Patients with T2DM show endothelial injury and dysfunction, which were worse in patients with T2DM and PE. High sTM and vWF levels have certain clinical predictive values for screening T2DM accompanied by PE.

## Background

Pulmonary embolism (PE) represents the mechanical obstruction of one or more branches of the pulmonary vasculature, usually due to a blood clot (thromboembolism) from deep vein thrombosis (DVT) [1–3]. Less common types of PE include septic emboli, venous air emboli, tumor emboli, and fat emboli [1–3]. The annual incidence of PE is about 60 per 100,000, although it may be lower in Asian populations [4]. The complications of PE include arrhythmia, chronic thromboembolic pulmonary hypertension, and cor pulmonale, which may lead to obstructive shock [1–3, 5]. Consideration for the prevention of venous thromboembolism (VTE), including PE, is critically important in both medical and surgical patients, mainly because VTE and PE lack typical clinical manifestations, especially in surgical patients, and can even manifest as sudden death [6–8]. With the deepening of clinicians’ understanding of PE, the diagnosis rate of PE is increasing [9]. PE has become the third largest cardiovascular and cerebrovascular disease in China after coronary artery disease and stroke, with a high risk of sudden death and a poor prognosis [10, 11].

The risk factors for VTE (including PE) include prior VTE, thrombophilia, surgery, cancer, pregnancy, immobilization, trauma, obesity, and central venous access [4, 12]. PE shares risk factors with other arterial diseases, especially atherosclerosis, including smoking, obesity, hypercholesterolemia, hypertension, and diabetes [4, 12]. The prevalence of type 2 diabetes (T2DM) in people over 18 years old in China has risen to 12.4% [13], even reaching 18.8% in older Chinese adults [14]. Hyperglycemia in T2DM is associated with macrovascular (atherosclerosis, coronary artery disease, and stroke) and microvascular complications (retinopathy, nephropathy, and neuropathy) [15–17].

Still, the relationship between T2DM and PE remains poorly understood. The hyperglycemic state in T2DM can lead to an imbalance of the fibrinolysis and coagulation system, and a hypercoagulability state is prone to lead to lower extremity DVT and PE [18]. T2DM is associated with oxidative stress, endothelial dysfunction, and low-grade inflammation [19, 20]. Vascular endothelial injury is involved in PE, and vascular endothelial dysfunction is the driving factor of atherosclerosis [21]. It is speculated that vascular endothelial injury and dysfunction could be the core factor of T2DM complicated with PE [22].

Soluble thrombomodulin (sTM) and von Willebrand factor (vWF) can be secreted by vascular endothelial cells, and their secretion will increase in the presence of endothelial cell injury [23, 24]. Circulating endothelial cells (CEC) are vascular endothelial cells that detach from the basement membrane and enter the blood due to injury caused by aging, hypoxia, inflammation, and other factors [25]. Under the pathological state, CECs undergo changes in number and morphology, and their count can be used as a marker of the damage level of diseased vessels [25].

Detecting the levels of vascular endothelial injury markers, including sTM, vWF, and CEC, could help unravel the relationship among T2DM, PE, and vascular endothelial dysfunction. Analyzing these relationships could help provide specific guidance for clinical diagnosis and treatment.

## Methods

### Study design and participants

This prospective study enrolled patients with T2DM hospitalized at Chu Hsien-I Memorial Hospital of Tianjin Medical University from January 2021 to June 2022. The participants were divided into the T2DM and T2DM + PE groups. The study was approved by the ethics committee of Chu Hsien-I Memorial Hospital of Tianjin Medical University (approval DXBYYkMEC2020-22). All participants signed the informed consent form.

The diagnosis of type 2 diabetes was based on the 1999 WHO diagnostic criteria [26]: 1) polydipsia, polyuria, polyphagia, and weight loss combined with random blood glucose ≥ 11.1 mmol/L; 2) fasting blood glucose ≥ 7.0 mmol/L or 2-h oral glucose tolerance test (OGTT) ≥ 11.1 mmol/L. The diagnosis of PE was based on the “Guidelines for the Diagnosis, Treatment, and Prevention of Pulmonary Thromboembolism” by the Pulmonary Embolism and Pulmonary Vascular Disease Group of the Respiratory Disease Branch of the Chinese Medical Association in 2018 [27]. All cases of PE were diagnosed by computed tomography (CT) pulmonary angiography (CTPA).

The exclusion criteria were 1) advanced malignant tumor, 2) congenital abnormal coagulation function, 3) pregnant or nursing women, or 4) lost follow-up or missing data.

The healthy controls were patients not meeting the diagnostic criteria for T2DM or PE. They were enrolled at the physical examination center of the hospital.

### CTPA

All participants underwent CTPA using a Philips Brilliance 64-row helical CT system. The scanning parameters were 488 mA, 120 kV, and 2-mm axial reconstruction layer thickness. Bilateral or single pulmonary artery filling defects were the basis for confirming PE.

### Markers

Fasting blood samples were collected from eligible T2DM participants in the early morning of the second day of admission, and fasting blood samples were collected in the early morning of the second day after diagnosing PE in patients. Fasting peripheral venous blood (12 mL) was collected in the morning from a cubital vein in citrate tubes. Nine mL of blood was mixed with 1 mL of citrate buffer and centrifuged at 3000 rpm for 10 min within 30 min of the blood draw. The supernatant was collected to obtain the plasma samples stored at -80 °C. sTM and vWF were determined using commercial ELISA kits (#542 h and #584 h, respectively; Anorikon Biotechnology Co., Ltd., Beijing, China) according to the manufacturer’s recommendations. The remaining 3 mL of venous blood was taken to determine the percentage of CEC by CD31^+^CD146^+^ flow cytometry (FITC anti-human CD31; #11–0319-41, Invitrogen Inc., Carlsbad, CA, USA; APC anti-human CD146, #361015, Biolegend, San Diego, CA, USA) on a NovoCyte 2000 analyzed (Agilent Technologies, Santa Clara, CA, USA). Glycated hemoglobin (HbA1c) was determined by a low-pressure liquid glycated hemoglobin analyzer (Sebia Minicap Flex Piercing, Sebia, France). Total cholesterol (TC), triglycerides (TG), low-density lipoprotein cholesterol (LDL-C), and high-density lipoprotein cholesterol (HDL-C) were determined by an automatic biochemical analyzer (AU5800, Beckman Coulter, Brea, CA, USA). D-dimer was determined using a coagulometer (CS-5100, Sysmex Corp., Tokyo, Japan).

### Statistical analysis

SPSS 23.0 (IBM, Armonk, NY, USA) was used for statistical analysis. The continuous data were expressed as means ± standard deviations. The continuous data with a normal distribution and homogeneous variance were analyzed by ANOVA; otherwise, they were analyzed using the Kruskal–Wallis test. The categorical data were expressed as n (%) and analyzed using the chi-square test or Fisher’s exact test. The correlation analyses were performed using Pearson’s correlation analysis. Two-sided *P*-values < 0.05 were considered statistically significant.

## Results

### Characteristics of the participants

The T2DM group (*n* = 30) included 18 males and 12 females, with an average age of 48.60 ± 15.71 years. The T2DM + PE group (*n* = 30) included 14 males and 16 females, with an average age of 66.13 ± 9.93 years. The healthy control group included 14 males and 16 females, with an average age of 37.60 ± 9.97 years. The participants in the T2DM + PE group were significantly older than in the T2DM and control groups (all *P* < 0.05), without differences for sex (*P* = 0.491) (Table 1).

**Table 1:** Demographic characteristics of the participants

	T2DM + PE group (*n* = 30)	T2DM group (*n* = 30)	Control group (*n* = 30)	X^2^	P
Age	66.13 ± 9.93^bc^	48.6 ± 15.7^ac^	37.6 ± 9.97^ab^	45.084	< 0.001
Sex				1.423	0.491
Male	14	18	14		
Female	16	12	16		

*T2DM* Type 2 diabetes mellitus, *PE* Pulmonary embolism

^a^ *P* < 0.05 vs. the T2DM + PE group

^b^ *P* < 0.05 vs. the T2DM group

^c^ *P* < 0.05 vs. the control group

### Comparison of laboratory indicators among the three groups

The FBG and HbA1c levels in the T2DM + PE and T2DM groups were significantly higher than in the control group (all *P* < 0.05), but there were no significant differences in FBG and HbA1c between the T2DM + PE and T2DM groups. The D-dimer levels in the T2DM + PE group were higher than in the T2DM and control groups (all *P* < 0.05). Compared with the T2DM and control groups, the patients in the T2DM + PE group were more likely to display hypoproteinemia and proteinuria. The creatinine levels in the T2DM + PE group were higher than in the control group (*P* < 0.05). The TC levels in the T2DM + PE group were lower than in the T2DM and control groups. Compared with the T2DM and control groups, the TG levels were higher and the HDL-C levels lower in the T2DM + PE group (all *P* < 0.05). The PaO2 in the T2DM + PE group was lower than in the T2DM and control groups (all *P* < 0.05). There were no significant differences in PaCO2 (all *P* > 0.05) (Table 2).

**Table 2:** Comparison of laboratory indicators among three groups

	T2DM + PE group (*n* = 30)	T2DM group (*n* = 30)	Control group (*n* = 30)	X^2^/F	*P*
FBG (mmol/L)	9.2 ± 3.51^c^	8.09 ± 1.49^c^	5.4 ± 0.34^ab^	48.464	< 0.001
HbA1c (%)	8.92 ± 2.77^c^	8.48 ± 2.38^c^	5.04 ± 0.63^ab^	52.838	< 0.001
D-dimer (mg/L)	3.12 ± 3.42^bc^	0.41 ± 0.42^a^	0.24 ± 0.08^a^	49.000	< 0.001
Albumin* (g/L)				10.588	0.005
> 30	25	30	30		
≤ 30	5^bc^	0	0		
Creatinine (umol/L)	73.04 ± 22.7^c^	61.01 ± 9.73	59.1 ± 7.23^a^	9.145	0.010
Proteinuria#				41.918	< 0.001
+	17^bc^	0	0		
-	13	30	30		
TC (mmol/L)	1.91 ± 0.9^bc^	4.88 ± 1.2^a^	4.52 ± 0.83^a^	53.748	< 0.001
TG (mmol/L)	4.67 ± 1.57^bc^	1.41 ± 0.85^a^	1.11 ± 0.84^a^	56.358	< 0.001
HDL-C (mmol/L)	0.98 ± 0.3^bc^	1.19 ± 0.21^a^	1.27 ± 0.2^a^	F 11.162	< 0.001
LDL-C (mmol/L)	3.26 ± 1.19	3.29 ± 0.92	2.8 ± 0.8	F 2.297	0.107
PaO2 (mmHg)	71.95 ± 13.54^bc^	88.71 ± 6.15^a^	89.53 ± 3.87^a^	35.873	< 0.001
PaCO2 (mmHg)	36.0 ± 5.01	37.90 ± 2.8	37.76 ± 2.69	3.875	0.144

*T2DM* Type 2 diabetes mellitus, *PE* Pulmonary embolism, *FBG* Fasting blood glucose, *HbA1c* Glycated hemoglobin, *TC* Total cholesterol, *TG* Triglycerides, *HDL-C* High-density lipoprotein cholesterol, *LDL-C* Low-density lipoprotein cholesterol, *PaO2* Partial oxygen pressure, *PaCO2* Partial carbon dioxide pressure

^*^Albumin ≤ 30 g/L was defined as hypoproteinemia

^a^ *P* < 0.05 vs. the T2DM + PE group

^b^ *P* < 0.05 vs. the T2DM group

^c^ *P* < 0.05 vs. the control group

# 24-h urine protein ≥ 0.12 g was defined as proteinuria

### Comparison of vascular endothelial injury markers among three groups

**Fig. 1:**
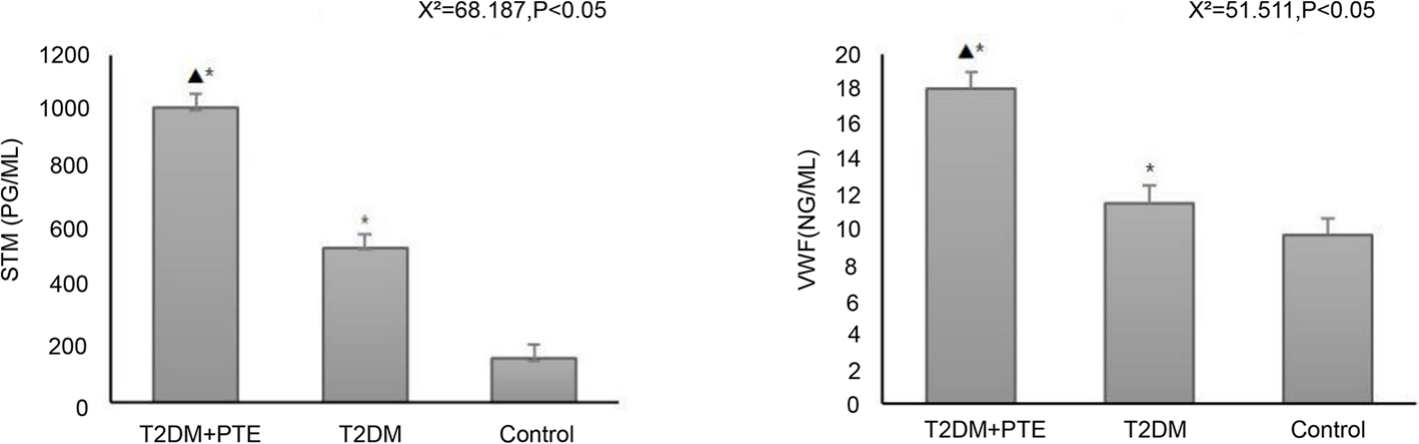
Comparison of levels of soluble thrombomodulin (sTM) and von Willebrand factor (vWF) in plasma among three groups. * *P* < 0.05 vs. the control group; ^△^ *P* < 0.05 vs. the T2DM group

**Fig. 2:**
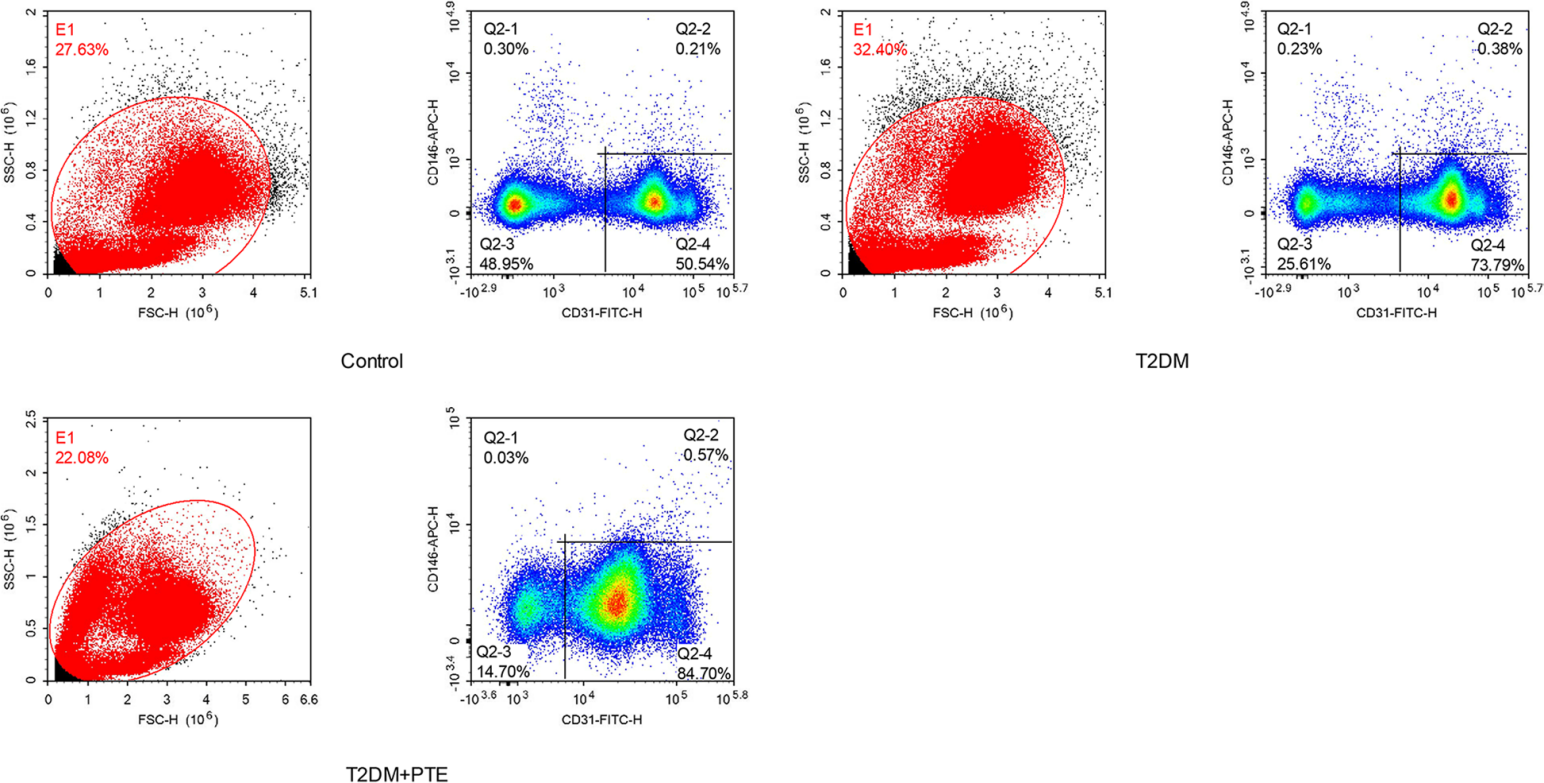
Percentage of circulating endothelial cells (CEC) (%) among three groups

sTM, vWF, and CECs were measured to determine the differences in endothelial injury among the three groups. The plasma levels of sTM and vWF and the percentage of CECs gradually increased from the control group to the T2DM group to the T2DM + PE group (all *P* < 0.05) (Table 3 and Figs. 1 and 2).

**Table 3:** Comparison of vascular endothelial injury markers among three groups

	T2DM + PE group (*n* = 30)	T2DM group (*n* = 30)	Control group (*n* = 30)	X^2^/F	*P*
sTM (pg/mL)	1016.51 ± 218.0^bc^	532.93 ± 243.82^ac^	151.22 ± 120.57^ab^	68.187	< 0.001
vWF (ng/mL)	18.02 ± 3.4^bc^	11.5 ± 2.17^ac^	9.63 ± 2.73^ab^	51.511	< 0.001
CEC (%)	0.56 ± 0.18^bc^	0.3 ± 0.08^ac^	0.17 ± 0.46^ab^	F 44.461	< 0.001

*T2DM* Type 2 diabetes mellitus, *PE* Pulmonary embolism, *sTM* Soluble thrombomodulin, *vWF* Von Willebrand factor, *CEC* Circulating endothelial cells

^a^ *P* < 0.05 vs. the T2DM + PE group

^b^ *P* < 0.05 vs. the T2DM group

^c^ *P* < 0.05 vs. the control group

### Analysis of the characteristics of the T2DM + PE group

As shown in Table 4, most patients in the T2DM + PE group (90.0%) had a history of T2DM. Six patients had other risk factors like postoperative, fracture, high-dose hormones, or acute myocardial infarction. Twenty-one (70.0%) had hypertension, 15 (50.0%) had coronary artery disease, and 12 (40.0%) had a history of stroke. Fourteen (46.7%) had chest tightness, breathing difficulty, and breath holding. Thirteen (43.3%) were classified as low risk, five (16.7%) as intermediate-low risk, and 12 (40.0%) as intermediate-high risk). Twenty-one (70.0%) showed hypoxemia, eight (26.7%) showed respiratory failure, and 12 (40.0%) showed hypocapnia.

**Table 4:** Characteristics of the T2DM + PE group

Characteristics	Subgroup	n
Duration of diabetes	New-onset	3
	Median duration of 9 years	27
Other risk factors	Postoperative (< 7 days)	2
	Fracture	2
	History of high-dose hormonal use	1
	Acute myocardial infarction	1
Complication	Hypertension	21
	Coronary heart disease	15
	Old cerebral infarction	12
	Hypertension + coronary heart disease	13
	Hypertension + previous cerebral infarction	9
	Coronary heart disease + previous cerebral infarction	7
	Hypertension + coronary heart disease + previous cerebral infarction	6
Clinical symptoms	Chest tightness/breath holding/difficulty breathing	14
	Chest pain	4
	Syncope/impaired consciousness	2
Stratification by PE risk	Low-risk group	13
	Intermediate-low-risk group	5
	Intermediate-high-risk group	12
	High-risk group	0
Blood gas analysis	Hypoxemia	21
	Respiratory failure	8
	Hypocapnia	12

### Correlation analysis between sTM and vWF in plasma and laboratory indicators

Pearson correlation analysis showed that the plasma sTM levels were positively correlated with HbAlc (*r* = 0.479, *P* < 0.01), D-dimer (*r* = 0.391, *P* < 0.01), and creatinine (*r* = 0.258, *P* < 0.01) and negatively with HDL-C (*r* = -0.318, *P* < 0.01) and PO2 (*r* = -0.464, *P* < 0.01). Plasma vWF levels were positively correlated with HbAlc (*r* = 0.371, *P* < 0.01), D-dimer (*r* = 0.497, *P* < 0.01), creatinine (*r* = 0.305, *P* < 0.01), and sTM (r = 0.624, *P* < 0.01), and negatively with HDL-C (*r* = -0.339, *P* < 0.01), and PO2 (*r* = -0.479, *P* < 0.01) (Table 5).

**Table 5:** Correlation analysis between endothelial injury markers (sTM and vWF) and laboratory indicators

	HbA1c	D-Dimer	Cr	HDL-C	PO2	PCO2	sTM	vWF
HbA1c	1							
D-Dimer	0.211*	1						
Cr	0.192	0.264*	1					
HDL-C	-0.286**	-0.325**	-0.287**	1				
PO2	-0.314**	-0.369**	-0.418**	0.322**	1			
PCO2	-0.120	-0.036	-0.150	0.346**	0.088	1		
sTM	0.479**	0.391**	0.258*	-0.318**	-0.464**	-0.146	1	
vWF	0.371**	0.497**	0.305**	-0.339**	-0.479**	-0.117	0.624**	1

*HbA1c* Glycated hemoglobin, *Cr* Creatinine, *HDL-C* High-density lipoprotein cholesterol, *PaO2* Partial oxygen pressure, *PaCO2* Partial carbon dioxide pressure, *sTM* Soluble thrombomodulin, *vWF* Von Willebrand factor

^*^ *P* < 0.05

^**^ *P* < 0.01

### Logistic regression analysis

sTM, vWF, and CEC were taken as the independent variables and T2DM + PE as the dependent variable. sTM (OR = 1.002, 95%CI: 1.002–1.025, *P* = 0.022) and vWF (OR = 1.168, 95%CI: 1.168–2.916, *P* = 0.009) were associated with T2DM + PE (Table 6).

**Table 6:** Logistic regression analysis

Markers	β	Standard error	Wold X^2^	*P*	OR	95%CI
sTM	0.013	0.006	5.272	0.022	1.002	1.002–1.025
vWF	0.613	0.233	6.886	0.009	1.168	1.168–2.916
CEC	2.270	2.670	0.000	0.993	9.676	0.000–3.592
PO2	-0.207	0.045	21.346	0.000	0.813	0.744–0.888

*OR* Odds ratio, *CI* Confidence interval, *sTM* Soluble thrombomodulin, *vWF* Von Willebrand factor, *CEC* Circulating endothelial cells

### ROC analysis of the vascular endothelial damage markers

**Fig. 3:**
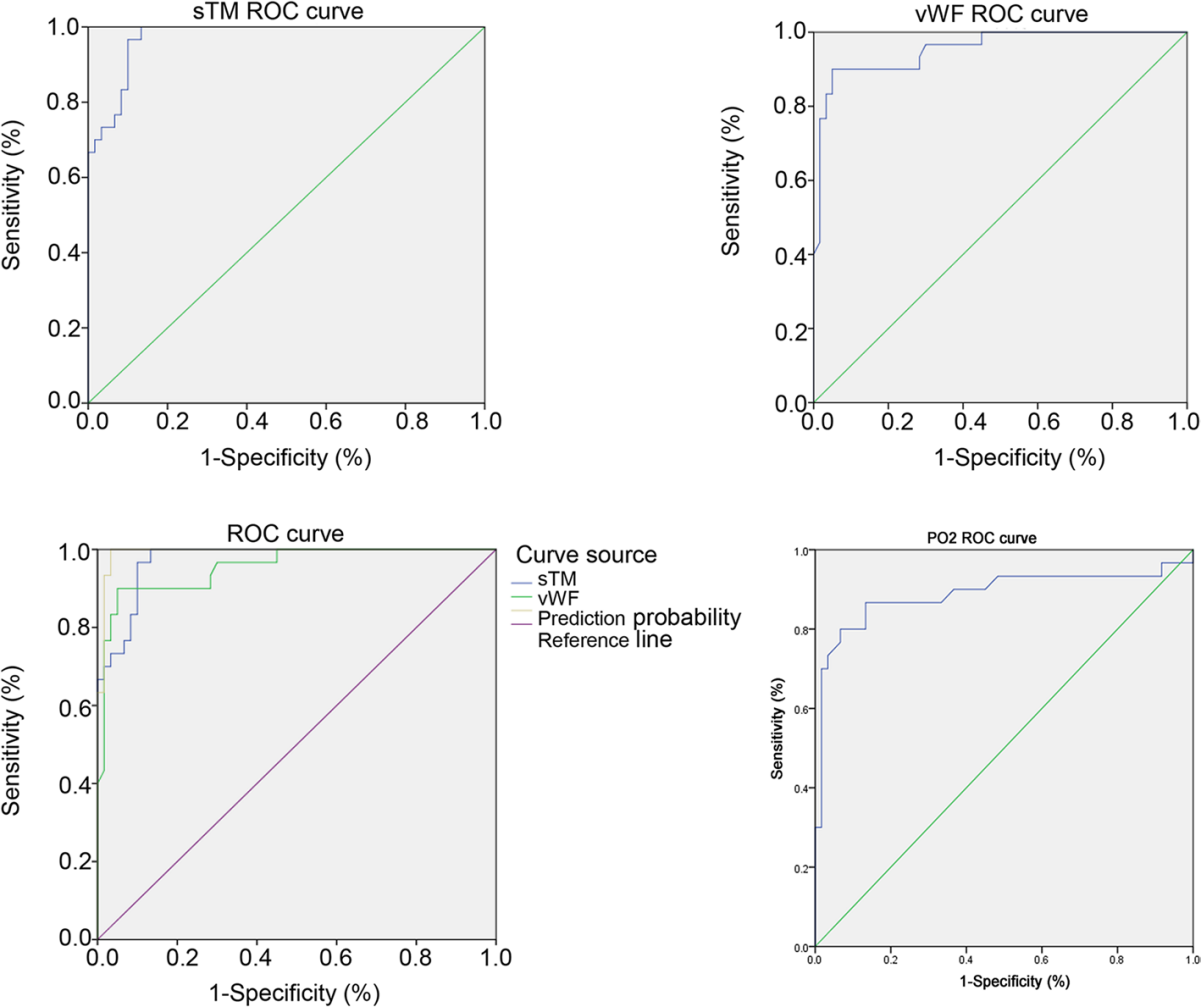
Receiver operating characteristics (ROC) analyses of vascular endothelial injury markers: soluble thrombomodulin (sTM) and von Willebrand factor (vWF)

Table 7 and Fig. 3 present the ROC curve analyses of sTM and vWF. sTM > 676.68 pg/mL for the diagnosis of T2DM + PE achieved an AUC of 0.973, with 100% sensitivity and 86.7% specificity. vWF > 13.75 ng/mL for the diagnosis of T2DM + PE achieved an AUC of 0.954, with 90.0% sensitivity and 95.0% specificity. The combination of both sTM and vWF above their cutoff points sTM achieved an AUC of 0.993, with 100% sensitivity and 96.7% specificity. At the same time, PO2 < 85.2 mmHg for the diagnosis of T2DM + PE achieved an AUC of 0.888, with 86.7% sensitivity and 86.7% specificity.

**Table 7:** Value of vascular endothelial injury markers (sTM and vWF) in screening T2DM + PE

Markers	Cutoff	AUC	*P*	Sensitivity (%)	Specificity (%)	95% CI	Youden index
sTM	676.68 pg/mL	0.973 ± 0.013	< 0.001	100	86.7	0.947–0.999	0.867
vWF	13.75 ng/mL	0.954 ± 0.022	< 0.001	90.0	95.0	0.911–0.998	0.85
sTM + vWF	-	0.993 ± 0.007	< 0.001	100	96.7	0.980–1.000	0.967
PO2	85.2 mmHg	0.888 ± 0.047	< 0.001	86.7	86.7	0.796–0.981	0.734

*AUC* Area under the curve, *sTM* Soluble thrombomodulin, *vWF* Von Willebrand factor, *CI* Confidence interval

## Discussion

Endothelial injury and dysfunction are involved in T2DM and PE, but the extent is unknown. This study investigated the changes and clinical significance of vascular endothelial injury markers in T2DM complicated with PE. The results suggest that patients with T2DM have endothelial injury and dysfunction, which were worse in patients with T2DM and PE. High sTM and vWF levels have clinical predictive values for screening T2DM accompanied by PE.

As early as 1856, Virchow et al. proposed three basic factors for PE: any factor that can lead to vascular endothelial damage, intravenous blood stasis, and hypercoagulability [28]. Hence, the involvement of endothelial dysfunction and injury in developing PE is not a recent concept. Still, a novelty is the exploration of common chronic diseases known to induce or exacerbate endothelial dysfunction and injury and their relationship with PE. It is well-known that T2DM is associated with vascular endothelial damage, coagulation dysfunction, and fibrinolytic dysfunction and that patients with T2DM are prone to ischemic stroke, myocardial infarction, lower limb thrombosis, PE, and other complications [22, 29]. In the present study, the results also showed that the levels of vascular endothelial injury markers were significantly higher in the T2DM + PE group compared with the T2DM group, indicating that vascular endothelial injury is involved in PE. TM is synthesized by vascular endothelial cells and attached to the surface of the cells, and is widely distributed in pulmonary blood vessels. After a vascular endothelial injury, TM is shed into the plasma to form sTM, and its concentration in plasma is significantly increased. Thus, sTM is an ideal marker of vascular endothelial injury [30]. vWF is a glycoprotein synthesized and secreted by vascular endothelial cells and macrophages. After vascular endothelial cell injury, endothelial cells are activated to release vWF, a marker of endothelial cell damage and dysfunction [24, 25]. The study also found that levels of sTM and vWF in the plasma of the patients with T2DM were higher than in the control group, and the levels of sTM and vWF in the T2DM + PE group were higher than in the T2DM group, indicating that patients with T2DM have endothelial function damage before vascular lesions and endothelial dysfunction was further aggravated in patients with T2DM + PE.

Circulating endothelial cells (CECs) line the inner wall of the blood vessels, and vascular endothelial cells can be detached from the basement membrane and enter the blood due to injury caused by aging, hypoxia, and inflammation. Under pathological states, CECs undergo changes in number and morphology, and their count can be used as a marker of the damage level of diseased vessels [25, 31]. In this study, the CEC counts were increased in patients with T2DM and T2DM + PE, suggesting gradually worse vascular endothelial injuries, but CEC was not associated with T2DM + PE in the regression analysis. Therefore, its predictive value was not analyzed by ROC curve analysis.

This study found that sTM and vWF correlated negatively with HDL-C levels and PO2. Indeed, HDL-C is well-known for its vasculoprotective effects through its involvement in reverse cholesterol transport and antioxidant properties [32]. PO2 is an index of blood oxygenation and is inversely related to PE [33]. Combined with the literature [33], the present study showed that the increase in sTM and vWF levels were positively correlated with the occurrence of PE and that they can be used as markers to predict the occurrence of PE. Our data show that the sensitivity and specificity of PO2 in predicting the occurrence of PE are 86.7%, while the sensitivity and specificity of sTM and vWE in predicting PE are as high as 100% and 96.7%, which are significantly higher than the predictive value of PO2. At the same time, the blood samples of sTM and vWF were venous blood, while arterial blood samples were needed to test PO2, which relieved the pain of blood collection to some extent. sTM and vWF were positively correlated with D-dimer and HbA1c in plasma. D-dimer in plasma was closely related to intravascular thrombosis and fibrinolysis [34], while HbA1c is a marker of overall glycemic control in the past 2–3 months [35]. Thus, it can be speculated that long-term hyperglycemia leads to vascular endothelial oxidative stress, impaired endothelial cell function, and exposure to intrasubcutaneous collagen fibers and activated endogenous coagulation pathways [36, 37], which promotes the development of PE. This study also indicated that attention should be paid to the compliance rate of HbA1c in diabetic patients, and vigilance should be necessary to avoid adverse consequences for patients with T2DM + PE due to long-term blood glucose control not meeting the standard and the interaction of several factors.

There is controversy regarding whether age is an independent risk factor for PE. Diabetes is an independent risk factor for PE and is related to age, according to a large population study [22, 38, 39], while some studies suggested that age is not associated with the occurrence of PE [40–42]. We found that the patients in the T2DM + PE group were significantly older than those in the T2DM group, which was statistically significant. T2DM mostly occurs in middle-aged and older adults. The various vascular and neurological complications of diabetes caused by long-term poor glycemic control are invisible and may take years or decades to develop into symptoms. Interestingly, the median history of diabetes in the T2DM + PE group was 9 years, suggesting that there is a possibility of PE occurring in patients with T2DM for about 10 years. Detecting vascular endothelial damage markers in these patients may help diagnose PE as soon as possible.

In this study, the predictive value of sTM and vWF for T2DM + PE was very high (AUC > 0.99). The results showed that the risk of PE was high when sTM was ≥ 676.68 pg/mL and vWF ≥ 13.75 ng/mL. sTM was predictive of PE in patients with COVID-19 [43] and patients after arthroplasty [44]. vWF is well-known for its high predictive value for future thromboembolic events [45, 46]. Still, the present study is the first to combine the two markers for predicting PE in T2DM. PE is notoriously difficult to diagnose because of a lack of specific signs and symptoms [9, 10, 28]. Therefore, measuring these two markers in hospitalized patients with T2DM could be a simple and effective way of determining the risk of PE. Future studies should also examine the same two markers in other patient populations. In addition to the endothelial damage markers we studied. We note that endothelial glycosylation degradation (syndecan-1[47]), endothelial cell activation (soluble endothelial selectin [se-Selectin]) [48] and endothelial cytotoxic histone (histone complex DNA fragment [hcDNAs]) [49] are also considered to be independent risk indicators for PE. Studies on other markers related to vascular endothelial injury and hypercoagulable state will be addressed in the future.

This study had limitations. The sample size was relatively small, and the results should be confirmed in larger cohorts. This study was cross-sectional, and no causality could be evaluated.

## Conclusions

In conclusion, vascular endothelial injury and dysfunction occur in patients with T2DM and T2DM + PE. Thus, monitoring the levels of vascular endothelial injury markers, such as sTM, vWF, and CEC, might be of certain guiding significance for the early identification of high-risk patients with T2DM + PE.

## Data Availability

All data generated or analysed during this study are included in this article.

## Abbreviations

T2DM: Type 2 diabetes mellitus
PE: Pulmonary embolism
sTM: Soluble thrombomodulin
vWF: Von Willebrand factor
CECs: Circulating endothelial cells
CTPA: Computed tomography pulmonary angiography
DVT: Deep vein thrombosis
VTE: Venous thromboembolism
OGTT: Oral glucose tolerance test
CT: Computed tomography
TC: Total cholesterol
TG: Triglycerides
LDL-C: Low-density lipoprotein cholesterol
HDL-C: High-density lipoprotein cholesterol
